# Hospital Meetings

**Published:** 1906-03-31

**Authors:** 


### HOSPITAL MEETINGS.

### MOUNT VERNON HOSPITAL FOR CONSUMPTION.

Sir Benjamin L. Cohen, Bart., took the chair at the annual meeting of Governors, held at the offices in Fitzroy Square on March 22.

The adoption of the annual report was moved by the Chairman, who said that, having read the report, he thought it would move anybody who gave it an attentive perusal. He wished most heartily to support the appeal for funds that was being circulated with the report. That appeal should arouse every sentiment and every instinct that should animate, not only those present, but all those acquainted with hospital management in London, and who knew the need for hospital treatment of the many sick poor in London. It should appeal to the benevolence of all right-minded people and also to the humanity of mankind. They would find in the report that 100 beds were still vacant, and he maintained that to keep those 100 beds out of occupation was, if not wilful murder, at any rate responsible homicide. A serious responsibility attached to the inhabitants of London for allowing those beds to be unoccupied, and thereby permitting many sick persons to succumb to disease whose lives might otherwise have been saved if they could have received necessary treatment. This was a responsibility from which humanity at large ought to recoil. Looking back over the last fifteen years, he said the managers of the hospital ought to be encouraged by the success that had rewarded their efforts so far. They owed a deep debt of gratitude to their President, Lord Zetland, for his splendid services to the hospital, and also to Dr. B. O'Connor, whom they were to elect as Honorary Governor as a small recognition of his work in the hospital for the last 25 years.

The resolution was seconded by Colonel Arthur and passed, and other business having been disposed of, Mr. Henry Stedall, Chairman of the committee of management, proposed a vote of thanks to the Chairman. He wanted, he said, to congratulate the Governors on the fact that Dr. Clifford Allbutt had consented to join Sir Hermann Weber as consulting physician to the hospital. With regard to finances, one of the members of the committee, Mr. Herbert Marnham, a great friend of the late Mr. B. Lyon, had given them £5,000 towards paying off the debt on the buildings at Hampstead. This splendid gift, together with £900 from Mrs. Wharry, reduced the amount of the debt, as mentioned in the report, by nearly £6,000. A tablet to the memory of the late Mr. B. Lyon had been erected in the hospital. Careful study of the medical report would show that the work of the hospital was not merely to cure the body, but to instil into the minds of the patients habits of precaution, which, when they returned to their homes, would prevent the spread of the disease. Touching upon Sir William Broadbent's suggestion that the 2,000 empty beds in the fever hospitals might be utilised for the treatment of consumptive patients, he dismissed it as unlikely to be adopted, and undesirable. In their hospital they had between sixty and eighty beds absolutely furnished, only waiting for funds for their up-keep. In olden days they had taken patients suffering from every kind of chest complaint at their hospital. Then, as their space grew limited, they had been obliged to restrict it to patients likely to be cured rapidly. Now that they were able to send these on to Northwood, they had returned at Hampstead to their proper sphere of dealing with patients with diseases of the chest. In consequence of this their death-rate had gone up. At Hampstead the death-rate was 5.5 per cent., at Northwood 1 per cent. A fortnight ago, when paying a visit to Northwood, he found nearly all the patients sleeping in the open air, which showed how advantage was taken of the mild weather. It was very satisfactory to think that the staff were now all well and comfortably housed.

The senior physician, Dr. Squire, read a letter from a former patient who had spent nearly twelve months in the hospital and who, when admitted, seemed in such an advanced stage of consumption that there appeared little chance of arresting the disease. This man was now in Canada, leading a hardy, strenuous life and enjoying perfect health.

### CITY ORTHOPÆDIC HOSPITAL.

The annual Court of Governors was held at the hospital on March 21, the chair being taken by Mr. W. G. Brighten.

The adoption of the report was moved by Mr. E. Noble Smith and seconded by Mr. J. M. Klenck, who said that the utility of the hospital during the past year was fully shown by the fact that 216 in-patients had been admitted, necessitating 305 serious operations, whilst 1,302 new out-patients had been relieved, who made 5,500 attendances. These figures spoke well for the conditions and utility of the hospital, and served as a complete answer to the criticisms which had been made with regard to special hospitals. For upwards of fifty years this hospital had been carrying on its work for the relief of the crippled poor in the Metropolis and also from the provinces, and they were very glad to think they had been able to do so much good.

Mr. E. Noble Smith, in acknowledging a vote of thanks to the medical staff, said that an attempt had been made during the last two years to amalgamate this hospital with two others. They all felt very grateful to the King for the active interest he had taken in orthopædic surgery, but it was not to be expected that the Council of King Edward's Hospital Fund could be so thoroughly acquainted with the past history and circumstances of their hospital as they were themselves. They knew how it had been instituted and how at this end of London it was impossible for poor crippled children to get to the West-end. By far the greatest number of cases came from the East-end, and if this hospital was required fifty years ago, how much more was it needed now? While statistics showed that the greater number of patients came from the East-end it could be fairly maintained that the hospital was required in this central position. It should therefore be with great hesitation, if at all, any steps were taken to remove the institution from this part of London.

A vote of thanks to the chairman closed the proceedings.

### ROYAL DENTAL HOSPITAL OF LONDON.

The chair was taken at the annual meeting of this hospital, on March 15, by Mr. Daniel Hepburn, Vice-President.

The annual report having been adopted, the committee reelected, and various votes of thanks passed, the Chairman addressed the meeting. He gave a short sketch of the past history of the hospital, from the time of its establishment, nearly 48 years ago. In the first year of its existence over 2,000 patients attended the hospital. In 1874 it was necessary to find increased accommodation; and this was again the case in 1901, when the present hospital was opened, and it soon afterwards received the gracious patronage of the King. Nearly 100,000 operations were performed annually, about 50,000 of which took place under anæsthetics. Now that the work had increased so greatly it could only be efficiently carried on by the liberal help of the charitable public. Its management had never been called in question, and the fact that a liberal donation towards the liquidation of the debt had recently been received from King Edward's Hospital Fund was sufficient proof that it would stand the test of investigation and inspection. What was chiefly needed was a much stronger array of annual subscribers. He concluded by an urgent appeal for increased support.

The report shows the total number of patients to have been 27,730, their attendances having been 50,911. The total amount received by the General Maintenance Fund was £3,719, against £5,804 in 1904, the difference being chiefly accounted for by the legacies that had fallen in in the previous year, none having been received in 1905. The committee have to face loans from the bankers of £1,150, an overdraft to the bankers of £859, and £143 for outstanding accounts, and make an earnest appeal for funds.

### NEW HOSPITAL FOR WOMEN.

The new Hospital for Women always seems to succeed in attracting a good audience to the annual meeting, and the last occasion, on March 16, was no exception to this rule, the room being completely filled, whilst some remained standing throughout.

The chair was taken by Mrs. Humphry Ward, who moved the adoption of the report, and said that the enlargement of the hospital planned last year had now been completed, and eight new beds provided by the conversion of the servants' quarters into a ward; a new nurses' dining-room had been added, and the Nurses' Home enlarged to provide more accommodation for the nurses and to house the whole of the servants. The total cost had been £4,967. In response to a special appeal only £1,156 had been received. A great part of the balance had been met by the sale of investments, leaving a very small fund available for the general expenses of the hospital in case of any emergency. This was a state of things that did not commend itself to any responsible authority and ought not to be allowed to continue. She very much hoped that the response to the special appeal would be much greater and more satisfactory. She trusted the meeting would see to it that the funds of the hospital were not depleted in this way and that special efforts were made to provide for the special enlargements. There had been an increase of patients during the past year, 663 having been treated in the wards. The number of new out-patients was 7,138, and the total number of attendances amounted to 31,236. A second assistant-surgeon had been appointed last year, who bore the illustrious name of Garrett-Anderson (Miss Garrett-Anderson, M.D.). The work of the Pathological Department had much increased and was of the greatest assistance in the investigation of cases. Money was needed very badly for this department also. She felt great pleasure in coming there that day, as she had always taken a very special interest in the hospital. The work she had done at the Passmore Edwards Settlement, not far off, had caused her to look upon the hospital as one of the sources of help and comfort for the poor of that neighbourhood. Their Convalescent Home, too, was situated in that part of Hertfordshire where she had lived for many years. As one who had taken part in the great movement for women's education, she was deeply interested in the medical women's movement, which had had the hardest battles to fight in the past. But the doubts and anxieties that had been felt then were now completely dispelled. The objection had been raised that the movement would produce women with highly cultivated brains and without hearts. Anyone who went round the hospital and saw the way it was managed would realise how unfounded that idea was. The increased force of women's education had strengthened the power of their mental equipment without in any way interfering with the due development of their hearts and spirits.

The resolution was seconded by the Rev. E. F. Russell, who pleaded earnestly for the establishment of a children's ward.

The election of the managing committee was moved by Mr. P. W. Wilson, M.P., who said that he had paid a surprise visit to the hospital, and had been much struck with the quietness, order, and fresh air which dominated the building and seemed to speak of the good work going on there. Referring to the growing financial burden, he pointed out that unless the public wanted the State to step in and take over the hospitals private enterprise must come forward to provide sufficient funds for their support.

Mrs. Boyd, M.D., in seconding the resolution, remarked that it must not be thought they did nothing for children at that hospital. For some few years an out-patients' Children's Department had been established, and they also had a cot in each of the adult wards. They wanted money for many things, and the children's ward was a very big thing. Funds were needed for a Röntgen-ray apparatus, which would only cost from £20 to £30.

Mr. A. Gordon Pollock, Chairman of the hospital, proposed that the Rev. Prebendary Paget (Bishop-designate of Ipswich) be made a Vice-President, and, after alluding in terms of deep gratitude to the assistance Mr. Paget had rendered to the hospital for the past ten years, expressed the keen regret they all felt at losing him. The resolution was seconded by Miss Cock, M.D., and carried.

A vote of thanks to Mrs. Humphry Ward for taking the chair was moved by the Rev. Prebendary Paget, who, in the course of a very amusing speech, suggested that if at any future time Mrs. Humphry Ward were to introduce medical women, students, and nurses into her novels, they would like to think it was the afternoon she had spent at the new Women's Hospital which had endowed her descriptions of them with a touch of vivid reality.

Mrs. Humphry Ward, in reply, disclaimed all intention of introducing these figures into any book, as they would require the background of London, and that she felt was too vast a subject for her to touch upon.

### BRITISH LYING-IN HOSPITAL.

The 156th annual meeting of the British Lying-in Hospital, Endell Street, was held on the 22nd inst., Mr. Charles Farmer, chairman of the board of management, presiding. In moving the adoption of the report and accounts, he said that during 1905 the in-patients numbered 484, as compared with 449 in the previous year, while 665 out-patients were treated, as compared with 618 in 1904. He thought it a subject for congratulation that they had treated over 1,100 poor women that year without a single death. Turning to the accounts, it would be seen that the debit balance of £844 at the end of 1904 was now £1,219. True, they had had a donation of £120 since the beginning of the year, but if they did not get a substantial sum from King Edward's Fund they would have to go to the ladies and get them to take steps to reduce that deficit. As showing that the hospital was economically managed, it would be seen that though there had been a considerable increase in the work done, the increases in expenditure were small, many items remaining practically the same. The deficit of nearly £5,000 in regard to the building of the nurses' home had been very little reduced; they still hoped some kind, charitable person would come along and help them there. They had taken £270 less in pupils' fees, but he was glad to say they were coming in now at the rate of two or three a week. They had now 13, and they could only take 16. Against that loss they might set the increase of £210 in donations. It was surely remarkable that the cost of every inmate, nurses and patients, only worked out at one shilling each per day; no fault could be found on the score of extravagance, and that was largely due to their matron, who was a most excellent housekeeper. In conclusion, he would like to say that their Ladies' Association was of the greatest assistance in the work of the hospital, both with regard to the patients and pupils, and he was glad to see their Samaritan Fund had a balance of £55. Mrs. Strickland, the wife of their excellent Treasurer, was again organising one of her sales of hats. He understood that she went abroad and bought hats, and sold them here at a profit, turning her house upside down to do it. Not only did they get the immediate benefit, but that sort of thing got the hospital known and brought them new subscribers.

The resolution was agreed to, as was also the re-election of the President and Vice-Presidents, Treasurer, and Auditors. Mr. Chadwyck-Healey notified his retirement from the board owing to pressure of other work. Mr. Strickland, Mr. Nash, and Sir George Stirling, the retiring members, were re-elected, and the proceedings terminated with a vote of thanks to the Chairman.

